# The use of Open Reading frame ESTs (ORESTES) for analysis of the honey bee transcriptome

**DOI:** 10.1186/1471-2164-5-84

**Published:** 2004-11-03

**Authors:** Francis MF Nunes, Valeria Valente, Josane F Sousa, Marco AV Cunha, Daniel G Pinheiro, Rafaela M Maia, Daniela D Araujo, Maria CR Costa, Waleska K Martins, Alex F Carvalho, Nadia Monesi, Adriana M Nascimento, Pablo MV Peixoto, Maria FR Silva, Ricardo GP Ramos, Luis FL Reis, Emmanuel Dias-Neto, Sandro J Souza, Andrew JG Simpson, Marco A Zago, Ademilson EE Soares, Marcia MG Bitondi, Enilza M Espreafico, Foued S Espindola, Maria L Paco-Larson, Zila LP Simoes, Klaus Hartfelder, Wilson A Silva

**Affiliations:** 1 Departamento de Genética, Laboratório de Genética Molecular e Bioinformática, e Laboratório de Genética de Abelhas, Faculdade de Medicina de Ribeirão Preto, Universidade de São Paulo, Av. Bandeirantes 3900, 14040-900 Ribeirão Preto, SP, Brazil; 2 Centro de Terapia Celular e Centro Regional de Hemoterapia, Faculdade de Medicina de Ribeirão Preto, Universidade de São Paulo, Rua Tenente Catão Roxo, 2501, 14051-140 Ribeirão Preto, SP, Brazil; 3 Departamento de Biologia Celular e Molecular e Bioagentes Patogênicos, Faculdade de Medicina de Ribeirão Preto, Universidade de São Paulo, Av. Bandeirantes 3900, 14040-900 Ribeirão Preto, SP, Brazil; 4 Ludwig Institute for Cancer Research, São Paulo-Brazil; 5 Departamento de Análises Clínicas, Toxicológicas e Bromatológicas, Faculdade de Ciências Farmacêuticas de Ribeirão Preto, Av. do Café sn, 14040-903 Ribeirão Preto, SP, Brazil; 6 Departamento de Biologia, Faculdade de Filosofia, Ciências e Letras, Universidade de São Paulo, Av. Bandeirantes 3900, 14040-900 Ribeirão Preto, SP, Brazil; 7 Instituto de Genética e Bioquímica, Universidade Federal de Uberlândia, Av. Pará 1720 -Uberlândia 38400-982 MG, Brazil; 8 Laboratório de Neurociências (LIM-27), Instituto de Psiquiatria, Faculdade de Medicina – Universidade de São Paulo, Rua Dr. Ovidio de Campos, s/n – Consolação 05403-010, São Paulo, SP – Brazil; 9 Ludwig Institute for Cancer Research, 605 Third Avenue, New York, NY 10158; 10 Departamento de Clínica Médica, Laboratório de Hematologia, Faculdade de Medicina de Ribeirão Preto, Universidade de São Paulo, Av. Bandeirantes 3900, 14040-900 Ribeirão Preto, SP, Brazil

## Abstract

**Background:**

The ongoing efforts to sequence the honey bee genome require additional initiatives to define its transcriptome. Towards this end, we employed the Open Reading frame ESTs (ORESTES) strategy to generate profiles for the life cycle of *Apis mellifera *workers.

**Results:**

Of the 5,021 ORESTES, 35.2% matched with previously deposited *Apis *ESTs. The analysis of the remaining sequences defined a set of putative orthologs whose majority had their best-match hits with *Anopheles *and *Drosophila *genes. CAP3 assembly of the *Apis *ORESTES with the already existing 15,500 *Apis *ESTs generated 3,408 contigs. BLASTX comparison of these contigs with protein sets of organisms representing distinct phylogenetic clades revealed a total of 1,629 contigs that *Apis mellifera *shares with different taxa. Most (41%) represent genes that are in common to all taxa, another 21% are shared between metazoans (Bilateria), and 16% are shared only within the Insecta clade. A set of 23 putative genes presented a best match with human genes, many of which encode factors related to cell signaling/signal transduction. 1,779 contigs (52%) did not match any known sequence. Applying a correction factor deduced from a parallel analysis performed with *Drosophila melanogaster *ORESTES, we estimate that approximately half of these no-match ESTs contigs (22%) should represent *Apis*-specific genes.

**Conclusions:**

The versatile and cost-efficient ORESTES approach produced minilibraries for honey bee life cycle stages. Such information on central gene regions contributes to genome annotation and also lends itself to cross-transcriptome comparisons to reveal evolutionary trends in insect genomes.

## Background

The honey bee, *Apis mellifera*, occupies a prominent place in biological research due to its social behavior, learning capabilities, haplodiploid mechanism of sex determination, and plasticity in phenotype (caste) and longevity. Thus, it is a model organism for classical and sociogenetic studies. In addition, bees drive a large-scale apicultural industry, and also generate important income in small-scale subsistence beekeeping. And finally, bees are of great economic and ecological relevance for their role as generalist pollinators.

The decision to include the honey bee amongst the current organisms for complete genome sequencing,  was, therefore, well founded, yet information on its transcriptome is still meager. When starting this study, little over 250 genes were annotated as partial or full length coding sequences, and only about 15,500 expressed sequence tags (mainly 5'-ESTs generated from a normalized bee brain cDNA library [1]) were available in public databases. Thus, even after sequencing of the honey bee genome will be completed a considerable transcriptome sequencing effort will still be required for unequivocal genome annotation, gene identification, and subsequent functional studies.

We used the ORESTES (Open Reading frame Expressed Sequence Tags) strategy to generate ESTs from different life cycle stages of the honey bee, such as appropriate for a genome annotation initiative. This strategy preferentially generates ESTs of the central, and thus most informative portion of the transcript [2], and frequently also identifies less abundant mRNAs [3]. The efficacy of the Open Reading frame ESTs strategy, in the context of an organism for which there is limited genomic information, has recently been demonstrated for *Schistosoma mansoni *[4].

This cost-efficient approach increased the already existent *Apis *EST database by 30% new reads. Of the 5,021 ORESTES, only 35.2% matched with previously deposited *Apis *ESTs. When assembled with the existent *Apis *ESTs in the NCBI database, the ORESTES sequences extended 66% of the mixed contigs. Together these data indicate that the ORESTES methodology could effectively complement the current efforts towards the definition of the *Apis *transcriptome.

## Results and discussion

### Honey bee Open Reading frame ESTs

We generated a total of 87 mini-libraries from the four major life cycle stages of honey bee workers (embryo, larva, pupa, adult) by the use of arbitrary primers and a low-stringency RT-PCR protocol [2]. From these libraries we obtained 5,021 sequences of appropriate standard quality (sequence > 100 bases; Phred 15) and with an average size of 373.9 bp. These sequences were deposited in the GenBank EST database (accession numbers CK628548 to CK633568). In the annotation pipeline, these were first submitted to BLASTN searches against *Apis mellifera *sequences deposited in the NCBI EST database (dbEST). At this step, 35.2% (1,769) of the validated sequences matched *Apis *ESTs (Table 1). In a subsequent step, a BLASTX comparison of the remaining sequences against the nr-NCBI database permitted the annotation of an additional 22.4% (1,123) of the honey bee ORESTES, while the remaining 42.4% (2,129) did not match any known sequence. This rather large set of ESTs that did not result in significant alignment with any sequence deposited in non-redundant databases contains candidates for novel honey bee genes.

**Table 1:** *Apis mellifera *Open Reading frame ESTs.

**Sequencing results**	**Number of reads**
**Total analyzed reads**	**5021**
- Embryos	1358
- Larval stages	720
- Pupae	1219
- Adults	1479
- Stage mix	245
**Local alignment matches**	
- *Apis *ESTs from dbEST	1769^a^
- *Apis mellifera *sequences in GenBank	16*^b^
- Genes of other organisms (orthologs)	1123^b^
- No matches in GenBank	2129
**Clusterization results**	
- Number of contigs	488
- Number of singlets	893
- Total number of clusters	1381

^a^BLASTN against dbEST using an E-score of 10^-30 ^as cutoff value; ^b^BLASTX against the nr database used <10^-15 ^as the cutoff. *This set is also represented by ESTs in the dbEST database. It is included here as additional information only and is not to be summed up with the other matches.

**Figure 1:**
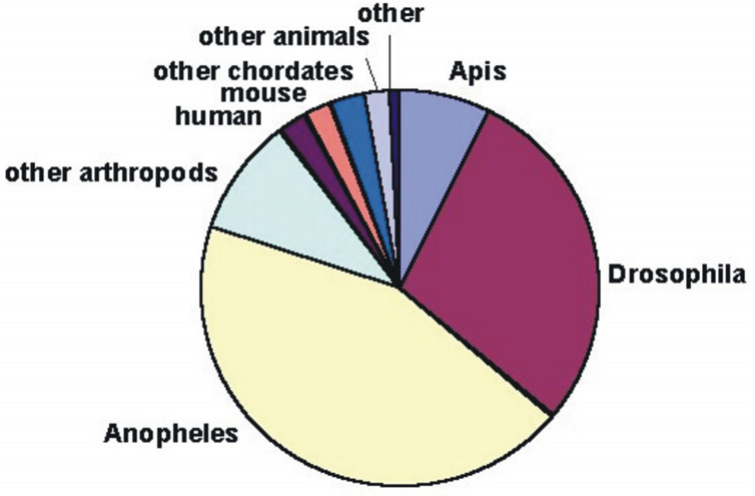
Distribution of Best-BLASTX-matches for assembled *Apis mellifera *Open Reading frame ESTs. After assembly into contigs and singlets the sequences were submitted to a search against a non-redundant protein database (NCBI). Independent of its E-score, the best match in each BLASTX result was listed according to organism category.

The 5,021 *Apis *ORESTES were assembled by CAP3 into 488 contigs of a mean size of 519 bp, leaving 893 singlets. In a second round of BLASTX comparisons against the nr-NCBI database, 28.5% of the contigs and 9.2% of the singlets were classified as putative orthologs. When the respective best matches were classified according to species or higher order taxa (Figure 1), 89.6% were from the arthropod clade (including fully or partially sequenced *Apis mellifera *genes). The largest fraction of these putative orthologs showed best matches with predicted *Anopheles *genes (43.9%), followed by ORESTES that were classified as putative orthologs of *Drosophila *(29.5%).

#### Gene Ontology classification

We assigned level 3 Gene Ontology (GO) classifications to 326 of the total of 488 assembled contigs; 162 contigs did not match any sequence in the nr-protein database. In the manual annotation preceding the GO analysis we preferentially assigned the contigs with respect to their *Drosophila *orthologs. The cellular component, biological process, and molecular function classifications of the honey bee sequences are shown in Table 2. In the biological process categories there is a clear prevalence for ESTs representing cell communication, cell growth and maintenance, metabolism and morphogenesis. For molecular function, the dominant assignments were to enzymatic activity and to nucleic acid binding and related functions (translation factor, transcription factor). When compared to the corresponding GO results obtained for the bee brain ESTs [1], we noted a similar distribution in category dominance structure, except for the molecular functions 'transporter and ligand binding/carrier' which have a higher representation in the bee brain ESTs than in our ORESTES contigs. This discrepancy most probably reflects functional differences in the tissues used in these two studies.

**Table 2:** Gene Ontology classification of *Apis mellifera *ORESTES contigs according to the *Drosophila *genes that they represent.

**Gene Ontology**	**Number of genes**
**Cellular Component**	
extracellular matrix	4
extracellular space	5
intracellular	99
membrane	29
others	8
**Biological Process**	
reproduction	18
cell motility	7
response to stress	6
cell communication	25
pattern specification	10
cell growth and/or maintenance	55
metabolism	79
response to external stimulus	10
morphogenesis	24
embryonic development	9
cell differentiation	9
others	41
**Molecular Function**	
nucleotide binding	14
nucleic acid binding	40
RNA polymerase II transcription factor activity	7
antimicrobial peptide activity	3
helicase activity	4
receptor signaling protein activity	5
structural constituent of cytoskeleton	5
microfilament motor activity	5
transcription factor activity	6
kinase activity	14
oxidoreductase activity	22
transferase activity	23
hydrolase activity	38
protein binding	29
metal ion binding	9
ion transporter activity	8
others	70

GO levels were set at 3. In either of the GO categories, individual contigs may be listed in more than one category. This GO classification only includes Apis mellifera orthologs to Drosophila genes that are represented by a Flybase code.

#### Clustering of honey bee ESTs

**Figure 2:**
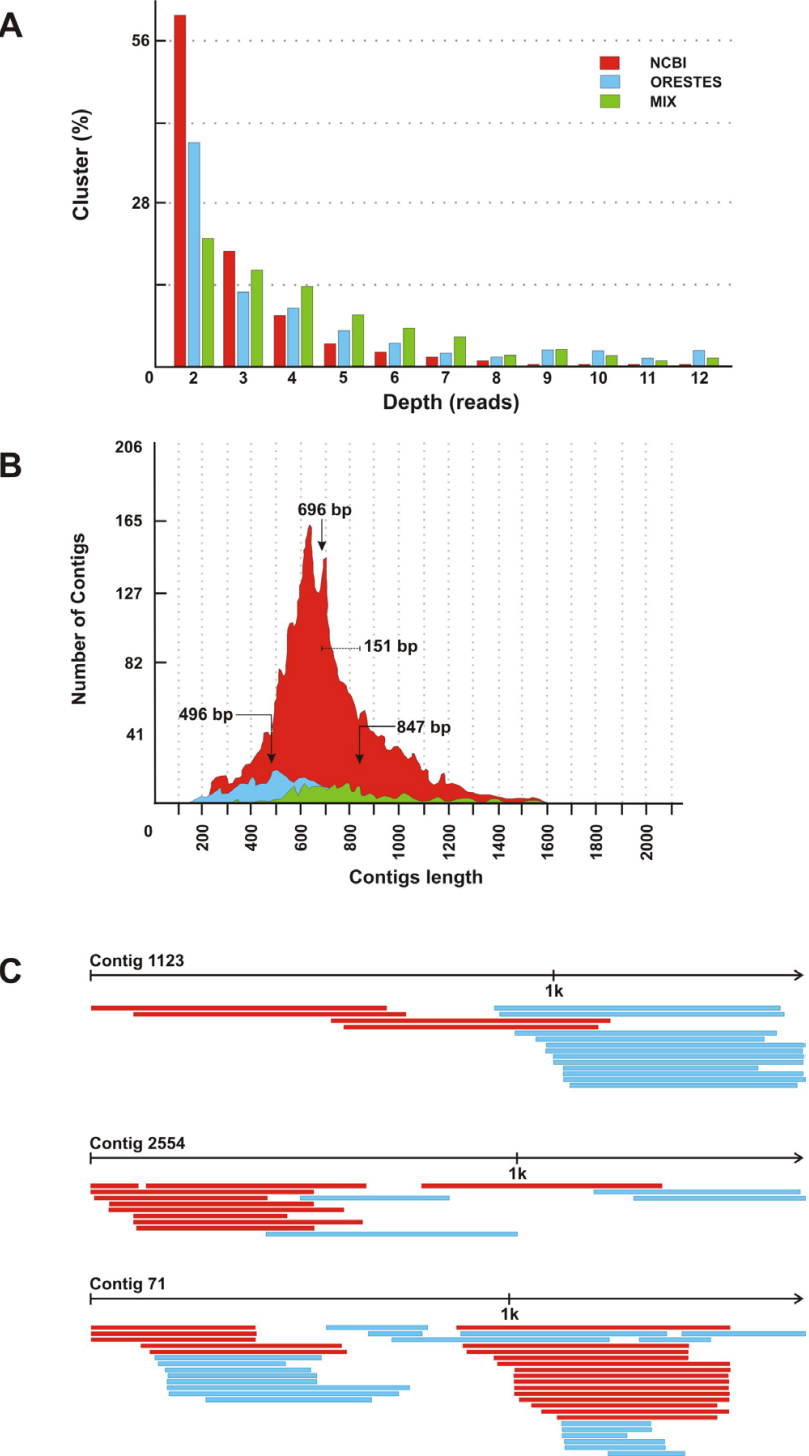
CAP3 assembly of *Apis mellifera *Open Reading frame ESTs (AmORESTES) with *Apis mellifera *ESTs previously deposited in dbEST (AmNCBI). A) Read depth distribution of pure AmNCBI or AmORESTES and of mixed contigs; B) EST size distribution of these contigs, C) Details of individual mixed contigs showing the extension and gap-closing characteristics. In all graphs, AmORESTES sequences are in blue, AmNCBI contigs are in red, and mixed contigs are in green.

We clustered the contigs generated in this study (AmORESTES contigs) with the *Apis mellifera *ESTs already present in the NCBI dbEST database (further referred to as AmNCBI contigs). Clustering performed by CAP3 resulted in a total of 3,408 contigs and led to a general increase in read depth (Figure 2A). This increase in read depth is reflected in the CAP3 assembled mixed sequences of the two databases. Mean length is 696 bp for the AmNCBI contigs and 496 bp for the AmORESTES contigs (Figure 2B). For the mixed contigs we noted a mean increase of about 150 bp in contig length, thus documenting that the ORESTES sequences add considerable information to the characterization of the honey bee transcriptome and for subsequent studies of specific genes.

Within the total contig population, 9.5% of the assembled sequences (323) are represented by mixed contigs of both AmORESTES and AmNCBI sequences, and within this group 66.3% (214) of the original AmNCBI contigs were considerably extended, or were joined across gaps by the AmORESTES contigs, as illustrated in Figure 2C. The fact that the number of mixed contigs is relatively low compared to total contig number may be attributed to two aspects. First, most of the AmNCBI contigs were obtained from a single tissue (brain) library, whereas the AmORESTES sequences represent whole body transcripts of all life cycle stages of the honey bee. Second, the AmNCBI sequences are mainly 5'-ESTs, whereas the AmORESTES sequences are expected to cover more central cDNA regions.

#### Genome comparison

Even though the total number of ESTs available for *Apis mellifera *is still low when compared to established genomic model organisms, we performed an across genome analysis with the set of 3,408 honey bee contigs. This involved sequential BLASTX searches, using the honey bee sequences as query entries against protein databases of *Drosophila melanogaster, Anopheles gambiae, Caenorhabditis elegans*, human, protozoan and fungal origin. With this selection of organisms we intended to extract information on the percentage of genes that *Apis *shares (i) with all organisms, (ii) with animals, (iii) with different sets of metazoans, (iv) and exclusively with insects. The cutoff E-value in these comparisons was set at 10^-6^, as used in comparisons of similar nature [4], and the representation of the respective putative orthologs was listed across taxonomic levels.

**Figure 3:**
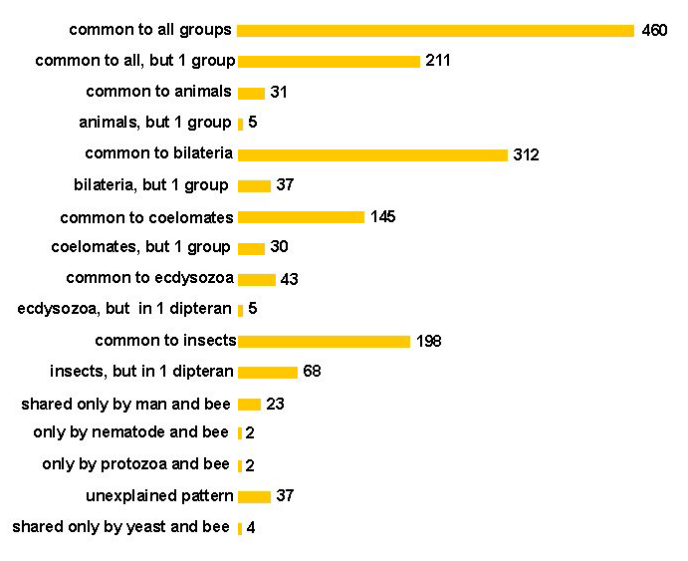
Similarity and representation pattern of assembled *Apis mellifera *ESTs (ORESTES + NCBI dbESTs) with predicted proteins of other organisms. In this comparison we included eukaryotes with completely sequenced genomes (*Drosophila melanogaster*, *Anopheles gambiae*, *Caenorhabditis elegans *and human), plus higher taxon groups, such as protozoans (primarily represented by *Plasmodium falciparum *and *P. yoelii*) and fungi (primarily represented by *Saccharomyces cerevisiae*, *Schizosaccharomyces pombe *and *Neurospora crassa*). These BLASTX comparisons were performed with an E-value cut-off level set at 10^-6^. Subsequently, the representation pattern of each of the *Apis *ESTs in each of the eukaryotic genomes was listed. Out of the total 3,408 *Apis *EST contigs, 1,629 could be classified as putative orthologs, and these were grouped according to the representation of these genes at the different taxonomic levels.

We found that 1,629 *Apis *contigs presented significant match with sequences belonging to at least one of the taxa genomes. From these, 460 contigs (28.2%) correspond to genes with a representation in all the above taxa (Figure 3). In addition, further 211 contigs (12.9%) could also be classified as common to all organisms since they were represented in all but in one of the members of this set of taxa (at this level, *Anopheles *and *Drosophila *were considered as a single group representing Diptera). This increases the set of EST contigs that the honey bee may share with all organisms to 41.2%, or, when considering the entire set of 3,408 contigs, to 19.7%. The second largest set of ESTs (312 + 37 contigs) is the one that is represented as genes common to the bilaterian clade (or metazoans in general), and only the third largest set (198 + 68 ESTs) contains genes that are represented solely in hymenopterans and dipterans, and thus in the insect clade.

Since deep-level phylogeny relationships within the bilateria are still a matter of debate, we separated our dataset according to the two prevalent hypotheses. The traditional view clusters arthropods within the coelomate clade. In our set of genomes, this tree architecture would be represented by genes shared between insects and the human genome. The alternative, more recently proposed hypothesis joins arthropods with nematodes to form an ecdysozoan clade [5]. The result of our comparison, which places emphasis on shared genes and not on the frequency of gene losses, is more consistent with the traditional view, since the coelomate clade is represented in this analysis with almost five times more shared genes than the ecdysozoan clade.

To infer on functional aspects within this pattern of genes that different clades appear to have in common we performed a Gene Ontology classification on biological process. In the set of *Apis *ESTs that stands for genes putatively shared with all organisms, the majority was classified as having a role in metabolism, and thus can be considered to represent basic functions. In contrast, the majority of *Apis *ESTs that are shared within the insect clade was represented in the biological process categories of cell growth and/or maintenance and cell communication. The corresponding insect-specific genes are therefore supposedly involved in more specialized functions. A similar conclusion can be reached from the micro- and macroarray analyses of transcripts detected in adult honey bee workers performing different tasks during their adult life cycle [6,7].

A total of 70 putative ortholog ESTs did not comply with any of the plausible phylogenies, yet nevertheless, this set may contain ESTs of interesting information content, especially when considering that the main set of genes within this group consists of *Apis mellifera *contigs that overlap with a mammalian genome. A manual analysis of these 23 contigs by BLASTX against the nr-NCBI database revealed that they are (at least by three orders of magnitude in E-values) more similar to mammals (especially to humans) than to other vertebrates and even other insects. This suggests that these genes may have diverged less in *Apis *and mammals and, therefore, may be subject to related selection pressure. Alternatively, at least some of them may have been modified in Diptera, and thus would show up as insect genes only once further non-dipteran insect genomes or transcriptomes have been sequenced and annotated. As shown in Table 3, this set of bee/human contigs contains a considerable number of predicted proteins related to cell signaling/signal transduction and transcription factors. Such a bias to information processing in our dataset of genes shared between honey bees and a mammalian genome may reveal system properties related to complex functions.

**Table 3:** Annotation and Gene Ontology characteristics of 11 honey bee EST contigs sharing significant similarity with mammalian but not with other vertebrate or invertebrate sequences. In all cases, the best match was with human proteins. For these 11 out of 23 contigs we could retrieve functional information.

***Apis *EST contig**	**E-score value^*a*^**		**GO, Biological process^*b*^**	**Human LOCUS ID GenBank annotation**	**Additional information**
	**Human**	**Insect**			
437	2e-67	NA	without result	NM_019116: ubiquitin binding protein	ubiquitin-specific protease domain
663	3e-10	NA	without result	NM_182830: MAM domain	contactin 5; neural adhesion molecule
1081	5e-07	9.7	without result	NM_013041: RAB3A interacting protein (rabin3)-like1	guanin nucleotide exchange factor domain
1425	8e-27	NA	without result	NM_182565: hypothetical protein MGC29814	TBP-associated factor 4; TATA box binding protein
1674	4e-82	NA	without result	NM_172374: interleukin 4 induced 1	none
1953	7e-11	NA	without result	NM_024707: gem (nuclear organelle) associated protein	spliceosomal snRNP biogenesis
2807	8e-07	5e-04	regulation of physiological process	NM_138457: forkhead box P4	transcription factor activity
2896	4e-05	0.002	reproduction, metabolism	NM_004654: ubiquitin-specific protease 9	ubiquitin thiolesterase activity
3167	2e-27	7e-05	cell communication	NM_033046: rhotekin	signal transduction
3347	5e-26	7.5	without result	NM_014006: PI-3-kinase-related kinase SMG-1	involved in nonsense-mediated mRNA decay
3374	1e-10	0.18	cell communication,	NM_014035: sorting nexing 24	intracellular signaling cascade

^a^E-value of the contig alignment with human or known insect sequences, NA: did not show any alignment; ^b^Gene Ontology results on Biological Process, FatiGO level 3, ^c^additional information obtained from Entrez Gene – NCBI and GOA link (GOAnnotations@EBI – European Bioinformatics Institute).

Finally, we found that 1,779 (52,2%) of the assembled EST contigs did not match with any sequence of the analyzed organisms. Such a large proportion of *Apis-*specific contigs is likely to be an overestimate. As noted in a previous study [1], this might be partly due to technical problems, such as, sequencing of cDNA inserts consisting mainly of 3'-untranslated regions, the presence of unspliced intron sequences, cDNAs with a negative reading frame, or chimaeric cDNAs. However, the major portion of the *Apis*-specific contigs may have become classified as species-specific due to their relatively short ORFs. We performed an ESTScan analysis  on the *Apis*-specific contigs which detected ORFs in 56% of the assembled ESTs. These ORFs are, however, relatively short, with a mean ORF length around 280 bp. Short ORF length represents a notorious problem to alignment algorithms resulting in low match scores, and consequently, a more frequent classification of short ORF ESTs as species-specific transcripts. For the honey bee, this has been shown for the brain cDNA library where 84% of the ESTs with ORFs shorter than 450 bp were classified as species-specific, against 24% in the EST set that had ORFs larger than 450 bp [1].

In order to gain a general perspective on the representation of species-specific ESTs we also directed our attention to estimates obtained in whole-genome cross-species analyses. For instance, a figure of 18.6% of species-specific genes was ascertained for *Drosophila melanogaster *in a genome comparison which included *Anopheles gambiae *as the other insect representative [8]. Based on this information, and taking advantage of a set of *Drosophila melanogaster *ORESTES, generated in a parallel project, we calculated the frequency of *Drosophila*-specific ORESTES sequences to obtain a more realistic estimate on *Apis*-specific genes in relatively small sets of ESTs. In this analysis, a set of 5,000 CAP3 assembled *Drosophila *ORESTES (409 contigs) was submitted to sequential BLASTX searches against protein databases of *Drosophila melanogaster*, *Anopheles gambiae*, *Caenorhabditis elegans*, human, protozoan and fungal, as described for the *Apis *contigs. For comparison, this same analysis was also performed with *Apis *ESTs, using separately 5,000 AmORESTES (486 contigs) and 5,000 AmNCB (632 contigs).

**Figure 4:**
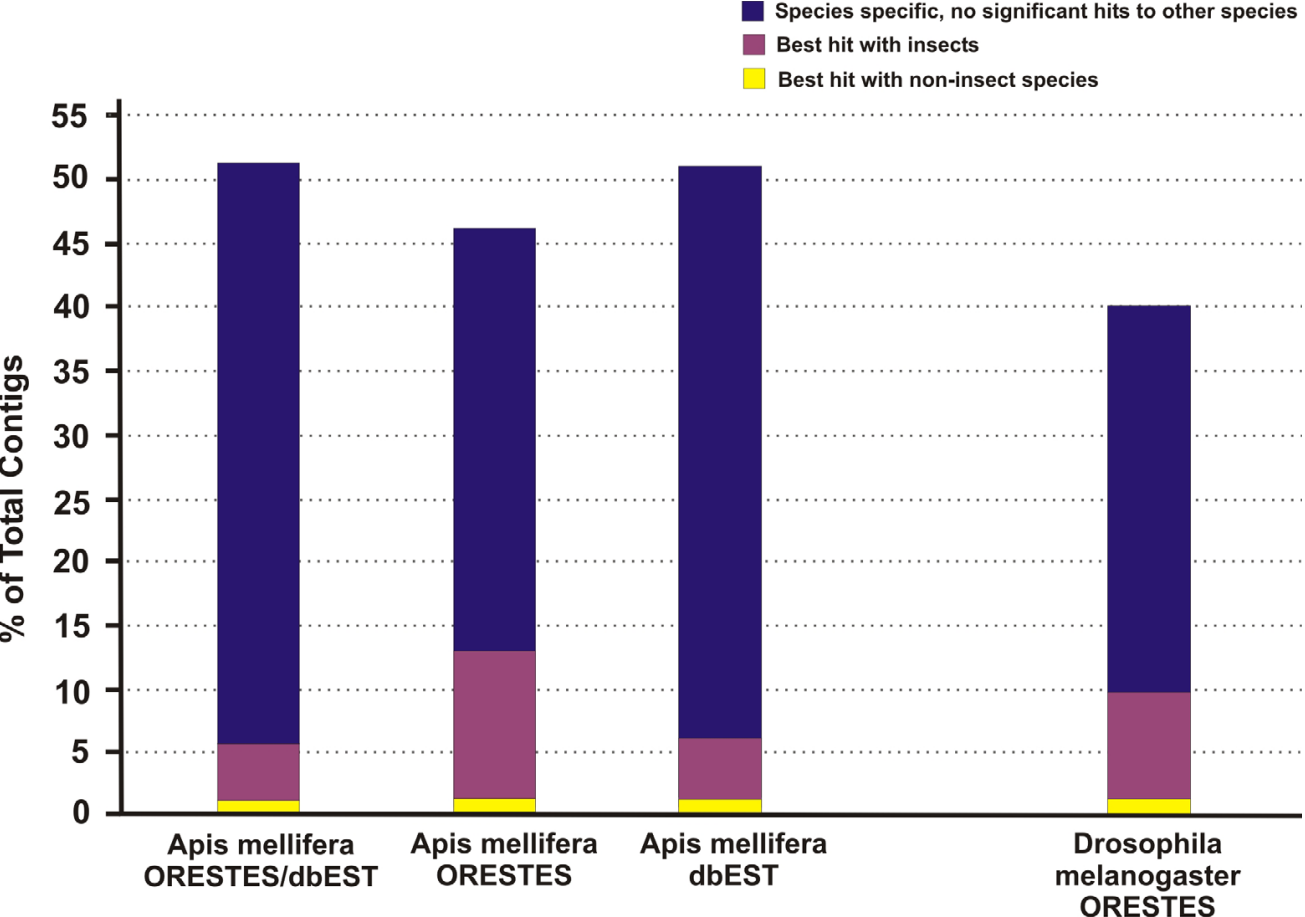
Percentage of honey bee and *Drosophila *ESTs representing putative species-specific genes (blue bars) in relation to ESTs that represent genes solely shared within the insect clade (pink bars), or that have higher similarity with eukaryotes other than the insect clade (yellow bars). In separate comparisons, the *Apis mellifera *contigs (ORESTES + NCBI dbESTs, n = 5,000), AmORESTES (n = 5,000), AmNCBI dbESTs (n = 5,000), and *Drosophila melanogaster *ORESTES contigs (n = 5,000) were analyzed against protein databases of an insect (*Anopheles gambiae*) and several non-insect species (*C. elegans*, protozoans, fungi and *H. sapiens*) with completely sequenced genomes. The cut-off E-value in these comparisons was set at 10^-6^.

The *Drosophila *and *Apis *EST contigs consistently showed relatively low proportions of insect-specific genes (6–13%). Still lower (ca. 1% each) was the proportion of ESTs that had significantly higher similarity scores with eukaryotes other than Insecta. In all EST sets we found a large fraction of sequences that were classified as either *Drosophila*-specific (40%) or *Apis*-specific (51% for AmNCBI dbESTs and 47% for AmORESTES). This high proportion of species-specific genes, therefore appears to be generated independent of the method used in EST sequencing, as it is represented in similar proportions in both the ORESTES set and the conventional 5'-EST set (Figure 4).

The figure of 40% *Drosophila*-specific genes obtained for our *Drosophila *ORESTES set can be directly set in contrast with the estimate of 18,6% species-specific genes reported in the *Drosophila *genome based study [8], and this would predict an overestimate factor of 2.15 for species-specific genes in the EST sets. When this factor is applied to the honey bee ORESTES, the 47% estimate for species-specific AmORESTES can thus be corrected to a more realistic figure of 22%. This estimate is in agreement with the results of Whitfield et al. [1] who observed that 24% of the honey bee genes represented by ESTs with ORFs larger than 450 bp did not have matches to any known protein sequences. This *Apis*-specific gene estimate is also in range when considering that the two dipteran species are thought to have separated from a common ancestor approximately 250 million years ago, whereas the postulated sister-group relationship of Hymenoptera and Mecopteroidea [9] suggests a pre-permian divergence, with a predicted separate lineage evolution of over 280 million years for honey bees and dipterans [10].

## Conclusions

The generation of a relative small set of Open Reading frame ESTs (ORESTES) that match and complement the already existent *Apis *EST database shows that this approach is sufficiently robust and favorably complements other strategies, such as ESTs prepared from normalized cDNA libraries. Its inherent properties of detecting transcripts of low abundance and aligning with central regions of transcripts [2,3] also make it a suitable tool in searches for novel honey bee genes and their annotation in parallel with ongoing genome sequencing projects. Furthermore, the genome comparisons performed in this and other studies [1,11] highlight that the elevated number of putative *Apis*-specific genes will still require extensive transcriptome sequencing for high quality genome annotation, and will play an important role in the question of insect genome organization and model systems in comparative studies [12].

## Methods

### Biological samples and RNA extraction

Samples of the four major stages of the honey bee life cycle were collected from *Apis mellifera *colonies (Africanized hybrids) kept in the experimental apiary of the Dept. Genetics, Univ. São Paulo, Campus Ribeirão Preto, Brazil. Each embryo sample contained approximately 300 eggs retrieved from a frame on which the queen had been caged for up to 72 hours. This assured that we covered the entire embryonic period. The larval sample was a representation of all five instars and included also spinning-stage larvae. Prepupae and pupae, including white-eyed, pink-eyed, brown-eyed and pigmenting pupae, were pooled into the pupal samples. For the adult sample we collected newly emerged bees, a random sample of hive bees (picked from a brood frame), and returning foragers. All these samples were snap frozen in liquid nitrogen. Total RNA was isolated using TRIzol reagent (Invitrogen). The lipid-rich larval and pupal samples required two additional extraction steps with phenol/chloroform and chloroform to obtain RNA of adequate purity.

In the case of *Drosophila melanogaster*, dechorionated embryos, larvae plus prepupae and pupae, as well as adult flies were collected from an isogenic *y*, *w*^1118 ^stock of *Drosophila melanogaster*. These were immediately frozen in liquid nitrogen and stored at -80°C until use. Total RNA was extracted with TRIzol, as described for *Apis mellifera*.

### Generation of Open Reading frame ESTs (ORESTES)

From high quality DNA-free total RNA samples we isolated poly(A)^+ ^RNA using an Oligotex II (Qiagen) kit. To assess poly(A)^+ ^RNA quality of the samples we performed Northern blot hybridizations with an actin (*Apis mellifera*) or tubulin (*Drosophila melanogaster*) probe. The probes were labeled by a random priming reaction in the presence of [α-^32^P]dCTP. The actin fragment was amplified using the primers described in Table 4. The *Drosophila *tubulin probe was already available from previous studies. High quality total RNA preparations were subjected to a DNase I treatment, and the absence of DNA contaminants was assessed by Southern blot hybridization of PCR products amplified with *Apis *or *Drosophila *16S mitochondrial DNA primers, respectively. High quality poly(A)^+ ^RNAs were aliquoted and stored at -80°C.

**Table 4:** Specific primers used to assess quality and absence of DNA contaminants of the RNA samples, and randomly selected primers used to generate cDNA profiles.

**Primer code**	**Sequence**
actin F (Apis)	5' AGCTATGAACTTCCAGATGGT 3'
actin R (Apis)	5' CCACATCTGTTGGAAGGT 3'
16S mitochondrial F (Apis)	5' TTATTCACCTGTTTATCAAAACAT 3'
16S mitochondrial R (Apis)	5' 'TATAGATAGAAACCAAYCTG 3'
16S mitochondrial F (Drosophila)	5' CCGGTCTGAACTCAGATCACGT 3'
16S mitochondrial R (Drosophila)	5' CGCCTGTTTAACAAAAACAT 3'
p3_2	5' TTGGGGATCGTATGTAGTATG 3'
pA82_1	5' CACTTCAGGATCCCTTGTAAGC 3'
pA82_2	5' CCAACATTGAATTCTCTTTGAC 3'
pA82_4	5' CAATAACAATGAATTCCAGAATCTCG 3'
pPT7C4_B	5' GCTTACAAGGGATCCTGAAGTGTTTCC 3'
pPT7C4_XS	5' GCAGGTAAACTCTACTCGAGTTACG 3'
M-RON-AS	5' CCAGGATGTTTGGGTGATGTA 3'
CREB-S	5' TCATGCAACATCATCTGCTCC 3'
H-SPARC-S	5' CTAACCCAAGACATGACATTC 3'
M-CD151-S	5' AAAGCTCGGAGGCAGCGAACT 3'
H-CD151-AS	5' CATGTGGCTGCAAGGCAAAGC 3'
M-SPARC-AS	5' GCCCAATTGCAGTTGAGTGAT 3'
M-ETS1-AS	5' GTCTTGATGATGGTGAGAGTC 3'
FUT-3-S	5' TCATGTCCAACCCTAAGTCAC 3'
FUT-3-AS	5' TCCAGCAGGCCTTGCAGAAAT 3'
M-CMET-S	5' TATCTCAAACGATCGAGAGAC 3'
M-CMET-AS	5' GCACATCTATTACCAGCTTTG 3'
H-CMET-S	5' TTTCAAATGGCCACGGGAC 3'
H-CMET-AS	5' GCACATTTATGACCATTCTCG 3'
H-Rhoc-AS	5' AGAAACAACTCCAGGGGCCTG 3'
M-Rhoc-AS	5' CTACCCAAAGCAGAAACCCCA 3'
H-Sparc-AS	5' CCAAAACCATCCTTGACAACA 3'
H-RON-AS	5' TGATGAGGTCCTTCACGGTG 3'
B237-2	5' CGGAATTCACCAGATTTGAACAGAAGAG 3'
B237-3	5' AACTGCAGTTAACCAGATTTGAACAGAAA 3'
GST_(PGEX)_NHE_I-S	5' CCGCTAGCATGTCCCCTATACTAGGTTA 3'
HOXA_I-F	5' CGCTCCCGCTGTTTACTCT 3'
P21-RasaI-F	5' GACCGCTCCTCCAACTAACC 3'
P21-RasaI-R	5' CCGGCCCACCTCTTCTACTA 3'
SRY8299.2	5' TCTCTTTATGGCAAGACTTACG 3'
SRY1532.1	5' TCCTTAGCAACCATTAATCTGG 3'
92R7.2	5' GCCTATCTACTTCAGTGATTTCT 3'
TAFIEX.1R	5' ATCCAAGGTTCTCCCAATA 3'

ORESTES profiles were generated according to Dias-Neto et al. [2]. Briefly, aliquots of 15 ng of purified mRNA were subjected to reverse transcription reactions utilizing SuperScript II Reverse Transcriptase (Invitrogen) and a set of randomly selected primers (Table 4). First-strand cDNA synthesis occurred at 37°C for 60 min in a total volume of 20 μl. The products of this reaction were diluted 1:5 in water and stored at -20°C. The cDNAs contained in 1 μl of each diluted RT-product were then amplified by PCR using the same or a single alternative random primer in a PCR mix (Ready-to-Go PCR bead, Amersham Biosciences). The amplification protocol consisted of an initial step at 75°C for 5 min, followed by a 45 cycles touchdown series (95°C for 30 s, a gradually decreasing annealing temperature from 66 to 44°C lasting 10 s per step and a decrease of 2°C per step, 72°C for 1 min), and a final extension reaction at 72°C for 7 min.

Aliquots of the PCR products (3–5 μl) were run on 1% agarose gels and stained with ethidium bromide. From profiles that presented near-even smears we excised two sets of amplification products, one covering a size range from 300 to 700 bp and a second one from 700 to 1500 bp. For cloning, these were extracted from the agarose gels (QIAquick Gel Extraction kit, Qiagen) and ligated into pUC18 (SureClone Ligation kit, Amersham Biosciences) for transformation of competent *E. coli *DH5α-cells by heat shock. Bacteria were grown in 2 × YT medium before aliquots were plated on 2 × YT agar containing ampicillin.

Blue-white selected positive colonies were picked, grown overnight in 2 × YT medium in 96-well plates, and used as templates for PCR using vector primers (M13 forward and reverse). An aliquot of each amplification product was analyzed on a 1% agarose gel before another 1 μl aliquot was submitted to DNA sequencing using standard protocols of the DYEnamic™ ET Terminator kit (Amersham Biosciences). The reaction products were analyzed in a MegaBACE™ 1000 automated sequencer. Only profiles with more than 80% positive PCR reactions were sequenced.

### Sequence analysis

After passing through the *Base Caller Cimaron 1.53 Slim Phredfy *(insert size > 100, "N" nucleotides less than 20%, and "N" repetitions of less than 6 nucleotides) and ScoreCard procedure (MegaBACE™) to check sequences quality, reads that were larger than 100 nt were submitted to an automated protocol for data analysis (Gene Annotation Pipeline) of the *Apis mellifera *or *Drosophila melanogaster *ORESTES. The protocol consisted of the following steps: conversion of electropherograms (Phred, to formats .fasta, .phd and .qual), primer and vector detection and trimming (Cross_match) and masking of repeats (RepeatMasker). Validated fasta format sequences were then submitted to a general BLASTN search against GenBank entries for mitochondrial and rRNA, as well as bacterial and fungal RNA to detect and eliminate contaminant sequences.

For the *Apis mellifera *ORESTES, subsequent BLASTN searches were performed against the approximately 15,500 *Apis mellifera *EST sequences deposited in GenBank dbEST. In this case, significant E values were set at 10^-30^. Searches against the non-redundant protein database entries used the BLASTX option with E-values set at 10^-10 ^as significance cut-off level.

CAP3 was used to clusterize the ORESTES sequences of both species. For *Apis mellifera*, the annotation of the 488 contigs was manually checked, giving preference to *Drosophila *sequences in the Unigene assignment. Subsequently, the contigs were batch submitted to a Gene Ontology procedure utilizing the FatiGO tools [13]. Clusterization of the *Apis *ORESTES contigs and singletons with the *Apis mellifera *ESTs deposited in GenBank dbEST was also performed using a CAP3 routine (standard parameters).

## Authors' contributions

*Apis *ORESTES: FMFN and JFS participated in all steps of library preparations data and analyses; MAVC and DGP performed the bioinformatics analyses; RMM, PMVP and MFRS participated in the library preparations and GO analysis; MCRC sequenced libraries; AMN performed validation PCRs on selected ORESTES; AEE participated in the design of the study and preparation of biological material; MMGB, EME, FSE and ZLPS participated in the design of the study, library preparations and conceptual data analysis; MLPL, VV and KH participated in the design of the study, library preparations and prepared the manuscript; WASjr coordinated the design of the study and the bioinformatics analysis.

*Drosophila *ORESTES: VV, JFS, DDA, RMM and EDN participated in all steps of library preparations and analyses; NM and RGRP participated in the design of the study and preparation of biological material; LFLR, WKM and AFC participated in RNA sample preparation; SJS, MAVC and WASjr participated in the design of the study and performed the bioinformatics analyses; AJGS, MAZ, EME and MLPL conceived and coordinated the study.

All authors read and approved the final manuscript.
